# Older persons’ knowledge of HIV and AIDS prevention in a province of South Africa

**DOI:** 10.4102/phcfm.v16i1.4264

**Published:** 2024-08-09

**Authors:** Sebastiana Zimba Kalula, Tarryn Blouws

**Affiliations:** 1 Department of Medicine, Faculty of Health Sciences, Groote Schuur Hospital, University of Cape Town, Cape Town, South Africa; 2 Department of Medicine, Faculty of Health Sciences, University of Cape Town, Cape Town, South Africa

**Keywords:** HIV and AIDS, knowledge, prevention, educational programmes, older persons, South Africa

## Abstract

**Background:**

Population ageing and access to antiretroviral therapy have resulted in an increase in the proportion of older people living with human immunodeficiency virus (HIV). However, scant knowledge is available to inform the design of educational programmes to target these persons in low- and middle-income countries.

**Aim:**

This study aimed to examine how persons aged ≥ 50 years view their risk of contracting HIV, and the extent to which they are supported in preventing infection and are impacted by the HIV or acquired immune deficiency syndrome (AIDS) epidemic.

**Setting:**

Rural sites in the Western Cape Province of South Africa.

**Methods:**

This study followed a qualitative design. Two focus group discussions with persons aged ≥ 50 years and interviews with two key informants were conducted at seniors’ centres. Discussions were digitally audio recorded and the recordings were transcribed, and data were thematically analysed.

**Results:**

Overall, awareness of the risk of older persons contracting HIV infection in this population was poor. Stigmatisation of the disease in the community and at health care facilities affected individuals’ willingness to be tested for the virus and/or to disclose their status, if positive. Participants viewed HIV and AIDS education programmes as focussed on the youth and educational sessions for large groups were not helpful in stemming the epidemic.

**Conclusion:**

Dissemination of information on older persons’ vulnerability to the disease, and education on HIV and AIDS tailored for and targeted at this age group have been relatively neglected.

**Contribution:**

Educational programmes on HIV, as well as productive channels and platforms to target older populations, particularly those with a low health literacy level are required.

## Introduction

Population ageing and increased access to treatment with antiretroviral therapies in South Africa are resulting in an increase in the proportion of older people living with HIV.^1,2,3,4^

The trend has implications for practice, policy and planning.^5^ Research studies on the HIV and AIDS epidemic have largely focussed on age groups up to 49 years.^6,7^ Human immunodeficiency virus and AIDS related research on older persons has primarily focussed on women as carers and surrogate parents to grandchildren orphaned after the loss of adult children to the disease. The older carers assume this role at a stage in their life when they themselves may have care needs.^8,9,10^ A gap in knowledge exists on how persons aged 50 years and beyond are affected by the epidemic other than this role. A low level of health literacy in older persons in rural communities in South Africa contributes to poor knowledge of HIV and AIDS.^11^

In 2016, 5.7 million (14%) of adults living with HIV were aged 50 years and older, and 65% live in sub-Saharan Africa (SSA).^4^ At the end of 2022, an estimated 37.5 million persons living with HIV globally were aged 15 years and older.^12^ By 2022, South Africa had approximately 8.45 million people (13.9% of the total population) living with HIV (PLWHIV),^13,14^ an increase from 5.38 million (10.6%) in 2011.^15^ A study in rural areas of South Africa on the prevalence of HIV in persons aged 50 years and older showed a rate of 16.5% (16.1% females, 17.7% males).^16^ Hontelez et al. project that HIV prevalence in South Africa will nearly double in 30 years, while the fraction of HIV-infected persons aged 50 years and older will triple in that period.^17^ Information is required on the support and education of persons aged 50 years and older on the risk of infection with HIV. Population ageing in the sub-continent is likely to exacerbate the existing HIV situation, with an increase in the number of older people living with the virus. Until now, relatively scant attention has been paid to older persons and the HIV and AIDS epidemic in SSA.^18,19^

The coronavirus disease 2019 (COVID-19) pandemic serves as a reminder moreover that older persons are the most vulnerable of all age groups to infectious diseases, including HIV, and associated poor outcomes, as shown by their heightened mortality in the COVID-19 pandemic.^20^ A major shift occurred in health care policy and practice in South Africa in 2019, during the COVID-19 pandemic, when priorities were redirected to fighting the COVID-19 pandemic.^21^ A focus on the prevention and treatment of COVID-19 led to neglect in the diagnosis and treatment of, and education on HIV and AIDS, further worsening the plight of vulnerable older persons.^21,22^

Widespread misconceptions exist about vulnerability to infection and sexuality in later life.^23^ Some older persons engage in high-risk behaviours, because of a lack of understanding of their vulnerability to contracting HIV. They are simply less likely than younger persons to view themselves at risk of infection, and less inclined or equipped to adopt preventive practices than other adults.^24^ In the African patriarchal context, older women are less able to negotiate safe sex with a partner than in some other cultures.^25^ Moreover, older persons have specific biological and psychosocial vulnerabilities to HIV infection.^26^ Older persons are not routinely screened for HIV by health care providers,^27^ and the disease is frequently misdiagnosed in its early stages. Besides, the disease is likely to be diagnosed only at an advanced stage and to progress more rapidly, because of lower age-related immunity and survival rates, than in younger adults.^5,28,29^ Despite these facts, older persons are seldom included in clinical trials.^30^

Challenges in the provision of education on HIV prevention to older persons therefore include ageism, older individuals’ reluctance to discuss sexuality and a misconception regarding their risk of contracting HIV. Misconceptions include older people being unlikely to change their behaviour, being neither interested in education and prevention programmes, and such programmes, therefore, being of little benefit.^31^

Available guidelines instruct health care professionals to share accurate information on HIV and AIDS with older clients to assess risk behaviours and to introduce strategies to reduce risk individually and in groups.^32,33,34^ However, health care providers themselves need to be educated on how best to approach older persons and present the information to increase awareness of HIV and AIDS in this age group.^31,35,36,37^

Knowledge about HIV transmission and prevention, accompanied by a reduction in risky behavioural practices are essential in combatting the spread of HIV among older persons. Hence, knowledge is needed of individuals’ perceptions of risk of infection and preventive practices in local populations.^38,39,40^ While the number of studies on older persons infected with HIV has increased in high-income countries, few studies have examined how HIV infection may be prevented in this age group in low- and middle-income countries. Knowledge is needed therefore to inform the design of interventions to target such persons, as well as to inform policy makers of the need for the interventions.

This qualitative study succeeded a large cross-sectional survey (N = 1163) which examined HIV and AIDS knowledge, attitudes and practices, and the health literacy of older persons in rural-dwelling populations at two sites.^11^ The purpose of the qualitative study was to explore the views of older persons on HIV and AIDS educational preventive support in depth, as well as perceptions of how older persons may be supported in preventing HIV infection. A key aim of the overall study was to provide evidence for the design of an educational intervention on HIV and AIDS for older persons. Participants in the qualitative study reported here did not participate in the quantitative survey.^11^

## Research methods and design

### Study setting

A qualitative research design was employed to collect data for the exploratory study. Sites for the study were the rural Cape Winelands district around Paarl and the town of Vredenburg on the West Coast, both in the Western Cape Province. The population aged 50 years and over of the Cape Winelands district constituted 16.9% and that of the West Coast district 18.6% of the respective populations of the districts. These districts have a higher proportion of the older population compared to the national average of 15.7%.^41,42^ A non-governmental organisation (NGO) in each study area assisted in the selection of the study site and the coordination of the research. The NGOs offer a venue at their seniors’ centre where persons come together to participate in activities and to benefit from services offered by the centre. The study population was persons aged 50 years and older resident in the areas, some of whom were a member of the senior centre in that area.

Non-governmental organisations set up and manage seniors’ centres in communities countrywide in South Africa. Some centres that meet certain criteria, such as evidence of a solid financial accounting system and the size of their membership, are subsidised by the government. Members pay a nominal membership fee and may attend a centre for 1–5 days a week. Centres offer a variety of activities, such as crafts, health education and physical exercises, as well as a midday meal. Some larger centres offer basic health screening by a nurse and some offer counselling by a social worker. A seniors’ club may be formed by members of a centre, which affords them an opportunity to socialise with peers while engaging in projects and income-generating activities such as food gardening. The proceeds of such activities are used to fund social outings and other needs.^11^

### Participant recruitment

The participants were purposively recruited^43^ at the centres with the assistance of the centre’s manager and a researcher (the second author, T.B.). The participants selected were those present at the centre on the day of the group discussion, and who were deemed by the centre manager and the researcher (T.B.) to be able to express themselves clearly and thus contribute thoughtfully to the discussion. In addition, the centre manager at each site, both females, were interviewed individually as key informants by the researcher (T.B.), using specially constructed interview guides aligned with the study objectives. The interview guides were piloted on three older persons to ensure clarity of the open-ended questions.

### Data collection

The focus group discussion technique was used to gather data from the participants. The two senior centres provided a quiet room in which the discussions were conducted. Two group discussions were conducted: one at each site. The size of the group in the Cape Winelands (Paarl) was nine persons (eight females and one male) and that in the West Coast district (Vredenburg) was five persons (four females and one male) (total *n* = 14). Membership of seniors’ centres tends to be predominantly female.^44^

Written, informed consent was obtained from each participant in a group prior to the start of the discussion and from each key informant prior to being interviewed. Discussions covered four main themes:

1. Knowledge about HIV and AIDS (sources of information, modes of transmission, symptoms and treatment);
2. attitudes towards HIV and AIDS (stigmatisation and awareness);
3. beliefs or perceptions about HIV and AIDS; and
4. practices to prevent contraction of the virus (protection).

Key informant interviews covered HIV and AIDS knowledge, practices and attitudes with a focus on the availability of educational activities that support and enhance these aspects as they relate to older people.

The group discussions were moderated by the second author (T.B.) who used a pre-constructed interview guide to pose questions and to facilitate discussion (see Online Appendix 1). T.B. had neither previous engagement with a study site nor with any of the participants, or with any health professional at the local primary health care facility. The level of subjectivity in moderating the discussions was thus minimised as follows: the discussions were conducted interchangeably in Afrikaans and/or English, the main languages spoken in the two communities, as preferred by a participant or the group. The moderator is fluent in both languages. The participants were encouraged to make input in the discussion and were asked for clarification of their input where needed. The moderator summarised what she understood from input and discussion, invited participants to correct any misinterpretation and encouraged further input before moving on to the next question. Data saturation was deemed to have been reached at the end of the second focus group discussion when no new topics were being raised. The key informants had face-to-face interviews in English using an interview guide (Online Appendix 2). The interview lasted 45 to 60 min.

The participants were encouraged to maintain confidentiality of the discussions, although such confidentiality cannot be assured in the case of focus group discussions. They were assured nonetheless of the confidentiality of all information they provided, and that at no time would their personal identity be divulged to any health care professional, nor be indicated in any publication. They were assured that the data would be kept in a secure place and only accessible to the researchers.

The research was conducted in accordance with the University of Cape Town’s Code of Ethics for Research guidelines. The group discussions and the key informant interviews were digitally audio recorded and the recordings transcribed. Both authors listened to the recordings before they were transcribed by T.B. Data recorded in Afrikaans, as provided by some participants, was translated into English and the translation was independently validated by Monica Ferreira, a gerontologist who is fluent in both languages and was not part of the study. Monica Ferreira, a gerontologist verified both the Afrikaans and the English translation of the transcripts. Written transcriptions were again reviewed by both authors. Once transcription was completed, the data were thematically analysed according to the pre-determined themes to create sub-themes.^45^

### Trustworthiness

The following criteria were adhered to ensure trustworthiness: credibility, transferability, dependability and confirmability.^46^ Data were obtained from two sources: older participants in the group discussions and the managers of the two senior centres which were the study sites. The study setting was within the community where the participants reside and access services, and where the managers provide the services to older persons. Data were collected from both the older participants and managers of NGOs providing services to older persons in the community. The main limitations of the study are the small rural sample size of predominantly female participants, who, compared to other older persons in the community may have been better informed on HIV and AIDs through exposure to activities at the senior centres.The researcher (Tarryn Blouws) who collected the data neither resides in the research community, nor is she a staff member of either of the health facilities. The researcher has experience in qualitative research.

## Results

The participants in the two focus group discussions had an educational level of seven or fewer years (≤ primary level education). The majority (73%) were beneficiaries of the means-tested social old age grant (the equivalent of US$113 per month in 2023), payable to eligible persons from the age of 60 years.

The participants articulated their views and level of understanding of HIV and AIDS, its impact on their community, and their perceived vulnerability to contracting the disease. The information set out below is categorised following thematic analysis. Data were analysed under the four themes:

1. Knowledge about HIV and AIDS;
2. attitudes towards HIV and AIDS;
3. beliefs or perceptions about HIV and AIDS; and
4. practices to prevent contracting the virus.

### Knowledge about HIV and AIDS

An overall concern articulated by the participants in the group discussions was the perceived huge impact the HIV and AIDS epidemic has had on their community and society. Knowledge of the disease in the older population of this community has been reported as being poor, however.^11^ Participants felt that people in their community only view certain groups as prone to becoming infected with the virus. People’s knowledge of the disease, some felt, could be improved through training workshops in the community to provide residents with accurate information. Some participants were sceptical of the effectiveness of educational talks offered to large groups and of information leaflets distributed door to door. A need for information about the disease, which includes information on modes of transmission and symptoms of HIV and AIDS, and treatment, was emphasised. Nonetheless, participants pointed out that their community has thus far seemed loathe to adopt and utilise such information.

#### Sources of information

Clinics and hospitals are the main sources of information on HIV and AIDS. Participants reported and pointed out that print and other media, for example, posters, television and radio are other sources. Community talks are yet another source of information, but some participants did not view them as educative, but rather as ‘street talk’, or gossip at a community level:

‘At the hospital it is hung up [*information posters*], that is where I see it.’ (61 year old, female, Paarl FG1)

‘She first heard about it in the community because they were speaking about this thing called HIV and they say it’s killing.’ (66 year old, female, Vredenburg FG2)

‘Old people don’t know about HIV and AIDS, they just see it on the TV.’ (64 year old, female, Paarl FG1)

‘On TV, because we cannot say we’ve seen someone [*have not had face-to-face interaction with an infected person*].’ (63 year old, female, Paarl FG1)

In addition, participants reported that an NGO in Paarl (Cape Winelands) and one in Vredenburg (West Coast) provide information to community members during door-to-door visits to render health care services. A key informant, who is a community health worker in Vredenburg, confirmed that:

‘Health care workers provide information while they do door-to-door service in the community, and distribute pamphlets provided by the clinic and the Department of Health who are our stakeholders in the health department.’ (54 year old, female, Vredenburg FG2)

A few participants in the focus groups confided that a person in their household is living with HIV and explained that family members are exposed to knowledge of the disease in this way. A participant spoke openly about her brother who was infected with the virus but now lives a full life because he is healthy, being compliant with his treatment:

‘When my brother also … he came out of prison and said he has AIDS. Do you know, that man is so healthy? That man who we thought would reach his sister’s grave … This brother who I would run after, we protected him. We walked with him on the path that he was on, we walked with him. And my brother is healthy, I am proud to say it because the Lord helped him.’ (59 year old, female, Paarl FG1)

A participant who disclosed her HIV status during a discussion shared her experience of living with the virus and shared that she applies her knowledge and experience to educate others. She contended that empowering others with first-hand knowledge of the disease is a positive role a person living with HIV can play:

‘Before I found out that I’m HIV positive, I was first tested and found that I got [*have*] TB. I was coughing a lot and I lost weight and the whole month I was going to the clinic. Myself, I took me because this fever just didn’t go away. In the second month I started losing energy then when I go to the clinic, they tested me and they found that I am also HIV positive.’ (65 year old, female, Vredenburg FG2)

#### Modes of transmission

Participants correctly reported that HIV can be transmitted through ‘unsafe’ sex and blood infected with the virus. Their explanation for ‘infected blood’ was when someone who is not infected has an exposed wound and is contaminated with blood from someone who is HIV-positive. Participants correctly articulated moreover that the virus cannot be transmitted through kissing, using a toilet used by someone with HIV or drinking or eating from the same cup or plate used by someone with HIV. They elaborated that it is important to wear gloves when in contact with someone’s blood, and that individuals, younger persons, in particular, should consistently use a condom during sex:

‘Yes, they say it’s when you have unprotected sex. Sometimes when someone has an open wound, through blood you can get it. They said you can’t get it when you [*are*] kissing somebody or using the same toilet or drinking from the same cup. You can’t get it. Only through blood and unprotected sex.’ (68 year old, female, Vredenburg FG2)

‘Injection with needles (referring to drug use, as a mode of transmission of the virus).’ (62 year old, male, Paarl FG1)

‘If you come into contact with someone’s blood.’ (67 year old, female, Paarl FG1)

#### Symptoms

Participants described symptoms experienced by an HIV-positive person as typically including sweating, fever, weight loss, coughing, headaches, loss of energy, skin pigmentation (skin becomes darker) and a compromised immune system:

‘… [*h*]e was coughing also. Also, there was a small scratch, and it grows and [*kept*] growing and every time he scratches but at the clinic before they didn’t see … Then it was becoming dark, his face. People I’ve seen with HIV, they [*are*] dark, they become very dark in colour. The mouth is like burnt, this I’ve seen.’ (66 year old, female, Vredenburg FG2)

‘… [*T*]hat child lay there, the fan is there by her, on her. She is boiling from the heat. Her eyes are this big … [*t*]hat child lay there, two fans on her but she is still sweating’ (61 year old, female, Paarl FG1)

Those participants who had seen an HIV-positive person at his or her ‘worst’ described such a person vividly:

‘The skull caves in.’ (61 year old, female, Paarl FG1)

‘She looks as if there is nothing of her body, almost like a skeleton from her head to her feet. It is just eyes that are dead still. She just looks at you, as if she is already dead.’ (61 year old, female, Paarl FG1)

‘The body withers away.’ (61 year old, female, Paarl FG1)

#### Treatment

A participant described how her son had died from the virus because he had not received ‘proper’ treatment:

‘Perhaps if he had had better treatment’, she stated, ‘[H]e would have lived a few months longer. However, he had not been compliant with his medication, and had neither changed his lifestyle at all.’ (68 year old, female, Vredenburg FG2)

The participants unanimously agreed that being compliant with treatment and living a healthy lifestyle are important. A healthy lifestyle is described as eating well, exercising and quitting unhealthy habits such as smoking and drinking alcohol:^47^

‘It [*medication*] works, yes, if you drink it, eat healthy and live healthily then it will work.’ (59 year old, female, Paarl FG1)

‘See now, like my brother – he eats healthily and looks after himself although he must attend hospital.’ (59 year old, female, Paarl FG1)

‘Difficult for some people to take these medications. Have to take [*medications*] for life, everyday many tablets. Better if one injection like for vaccine.’ (69 year old, female, Vredenburg FG2)

‘Why can’t they find a cure?’ (69 year old, female, Vredenburg FG2)

### Attitudes towards HIV and AIDS

Participants suggested that a societal shift in attitudes and mindsets towards individuals who are HIV positive is needed, for infected persons to feel accepted – in their community and in society. Although such a shift may be encouraged and promoted, it may not be widely accepted in a community, it was contended.

A key informant (in Paarl) pointed out that young persons who come to the clinic for family planning are not judged. Testing for HIV, she contended, must be available to everyone in a similar, non-judgemental way. A discussant reported disbelief when she had approached a clinic to be tested for HIV and was told she is too old to be tested:

‘I wanted to [*be tested*], but then they said “No, mam is too old.”’ (63 year old, female, Paarl FG1)

#### Stigmatisation

Participants perceived a notable lack of knowledge of HIV in their community. Although speculation abounds as to who in the community may be HIV positive, a fear of stigmatisation was identified as a possible reason for non-disclosure of HIV status. Participants viewed stigmatisation and non-disclosure as a negative aspect of the epidemic:

‘Nobody really says this one has AIDS or that one, they don’t know in our communities. That stigma is still there. That’s why people don’t want to open up.’ (64 year old, woman, Paarl FG1)

Discussants opined that people remain greatly affected by stigmatisation of the disease and as a result, few persons know who in their community is HIV positive. A participant described the fear of disclosure as crippling, as her friend was unable to speak openly about her status, not even to her family:

‘Long time ago he was having a friend, and the friend was having HIV, but then the friend was scared to tell the family. He was so scared for them to find out he is having HIV. But the problem he was facing in that time [*was*] he was losing weight and at the same time having a problem with the back waste [*diarrhoea*].’ (68 year old, female, Vredenburg FG2)

However, a participant who is HIV positive volunteered that she does not let fear of disclosing her status determine whether she can help people or not. She uses herself as an example – to bring awareness to others of this health issue:

‘I always speak about this, and my aim is to help you because when you come to me and say you suspect that you have a headache that doesn’t go away, or you have this pain, I advise them you must go to the clinic and also ask them to check if you have HIV.’ (65 year old, female, Vredenburg FG2)

Finally, reference was made to ‘a mindset of ignorance and a lack of understanding’ in that many individuals perceivably continue to view HIV and AIDS as ‘primarily affecting only certain groups (tied to a particular ethnic group):

‘You know when this HIV thing comes, people didn’t believe it is real. They just thought hai it’s this “Xhosa thing”, there is nothing like that. Maybe somebody died and then they didn’t clean you properly [*that is a bad omen due a cultural practice not followed*].’ (68 year old, female, Vredenburg FG2)

Hence, a contention is held at our study sites that the disease only occurs in a certain ethnic group.

#### Awareness

Participants reported that HIV and AIDS awareness programmes are offered in some communities. However, in general, they felt that the programmes were insufficient, or unproductive, and did not help to mitigate the spread of the epidemic in any notable way. Some stated that while certain organisations do run awareness campaigns, the community is not receptive to them. A key informant estimated that in a certain nearby community, awareness of the disease is ‘approximately 50 per cent’, and elaborated that a change of mindset is needed. In addition, the stigmatisation of individuals who are HIV-positive makes it more difficult to create awareness of the disease:

‘Rating (sic, presumably the statistical rate of awareness) very difficult, about 50%. Each training shows more people need to be educated regarding HIV and AIDS/STI, and their mindset should change, and stigma break down (sic) to up the rate.’ (51 year old, female, Key informant, Vredenburg)

Awareness programmes and campaigns should target people of all ages, not only the youth, participants explained – the exclusion of other groups such as older people, they viewed as being counter-productive. Education on the virus targeted specifically at older people may be a solution, some felt. Yet, it may prove difficult, as they previously mentioned, as older people may not be receptive to such education:

‘We must become more involved. We old people have already made a huge difference in the community. And especially our women. Women make a big difference, look in the churches and in the clubs. Previously you became old, got sick and died. But now we are in the clubs, and we come together.’ (62 year old, female, Paarl FG1)

Nonetheless, suggestions were proffered on forms that programmes and campaigns might take to target older persons. One form might be door-to-door interviews, as addressing (large) groups in clinics or at hall assemblies, some felt, is problematic (in Vredenburg, for example). People do not pay attention to the speaker, a participant explained; they lose interest in the topic or see the event rather as an opportunity to socialise with friends. In Paarl in the Cape Winelands, it was told that a group that works for a community clinic does monthly home visits to randomly selected older persons (clinic patients) and does HIV testing in their home:

‘They were there because the computer generated my daughter’s name, but she wasn’t there (at home at the time). So, they asked me if I didn’t want to be tested, so I did it.’ (61 year old, female, Paarl FG1)

‘I think door-to-door is much better because when you see them, sitting down talking, some will understand. When you are sitting one to one some will understand. When you call them to the hall and talk to them, they end up not even listening, they start making [*forming*] groups.’ (66 year old, female, Vredenburg FG2)

‘When they hear what she is going to talk about they [*people in the waiting area*] start talking immediately and they make a lot of noise, you can’t even hear what the person in front is talking about…They [*go*] off topic, they not even considering what is being said or close to what you talking about. Others will just walk out, or bored of [*disinterested in*] what you’re talking about.’ (66 year old, female, Vredenburg FG2)

A key informant stated that their organisation provides training for families to teach them how to care for members in their household who are ill as a result of the virus – and thus protect themselves from infection. This organisation integrates its services with those of other NGOs to offer workshops. The organisation engages trained care workers to do voluntary counselling and testing:

‘Our organisation is aware of HIV and AIDS workshops/services, we integrate with other NGOs and stay on [*the*] database of [*the*] health sector. Our organisation offers accredited health training.’ (51 year old, female, Key informant, Vredenburg)

### Beliefs about HIV and AIDS

All participants were knowledgeable that HIV cannot be cured: ‘Not by anyone or anything’, they stated. Not even traditional healers have the ‘power’ to cure this disease. A participant correctly explained: ‘It is an incurable disease but can be stabilised when using treatment consistently’.

People who do not believe that HIV exists, participants observed, are youth in the community. Such disbelief may be noted in their engagement in reckless behaviour (because of ‘external influences’), they elaborated. These persons are more susceptible to contracting the disease:

‘The youth of late are too entitled, [*e*]ven when clinic personnel go visit schools and schoolchildren can educate themselves about sex, our kids know too much. When you come with your condoms and stuff, they start laughing so they won’t take anything serious. You can give them condoms whatever, but it’s a joke to them. They not aware about this, how dangerous this thing is.’ (66 year old, female, Vredenburg FG2)

It is important for older people, participants continued, to understand that they ‘should not mistake that age excludes one from being infected’. An older man, it was explained, can be infected by a younger woman. Therefore, older people should be careful, particularly when it is older men and younger women (engaging in sexual intercourse):

‘You see, all the older people think they will never get that. But forget one day he take a younger lady and that younger lady is a problem. He himself staying with his old mummy, he won’t get that. But one day he is at the shebeen, anywhere, he finds the smaller one, the old mummy is at home. One day he takes that smaller one is bringing problem and he is older and say he won’t get that. So older one also need to be taught about it.’ (61 year old, female, Vredenburg FG2)

### Practices to prevent contracting the virus

Participants pointed out that HIV is ‘a big problem’ in communities where individuals engage in risky behaviour. Risky behaviour, they explained, includes misuse of alcohol and visiting ‘shebeens’ (taverns). Once intoxicated, some told, they lose inhibition and engage in irresponsible behaviour such as unsafe sex:

‘I think they simply don’t care.’ (57 year old, female, Paarl FG)

Participants were cognisant that practices to protect oneself from infection with the virus are having safe sex with the use of a condom, eating and living healthily, and wearing gloves when in contact with someone’s blood:

‘I think we should be careful of the blood.’ (63 year old, female, Paarl FG1)

‘If you want to help someone with blood around them, then you have to put on gloves.’ (68 year old, female, Vredenburg FG2)

Protection must come from both parties when engaging in sexual intercourse, some observed:

‘Now that people are getting treatment for HIV, people look good, you will never see that there’s fault. The people will never tell you, ‘I’ve got HIV’, so the better thing is to just use protection when having sex. People say you will never see this one having HIV because he is using treatment. He looks normal like everybody.’ (61 year old, female, Vredenburg FG2)

Overall, the discussants were of the opinion that there is some knowledge about HIV and AIDS in their community, but there are no concerted educational programmes in their community that are inclusive of and appropriate for older members. Older persons are assumed not to engage in sex and hence not to be at risk of HIV infection, and they are consequently marginalised in both educational and clinical programmes.

## Discussion

Our study set out to examine how people aged 50 years and older view their risk of contracting HIV, and the extent to which they are both supported, including in education programmes, and are affected by the HIV and AIDS epidemic in their community. Participants in the focus group discussions acknowledged that the HIV and AIDS epidemic has had a huge impact on their community, for both those infected with and those affected by the virus. Knowledge of HIV and AIDS has been widely reported to be poor in the older population.^11,48,49^ Some of our study participants had some basic knowledge of modes of transmission, symptoms and a need for compliance with treatment for HIV and AIDS. Similar to the findings of the study by Nyirenda et al.,^50^ the knowledge was largely from experience in a household with an infected family member or a study participant who was living with HIV.

Participants were particularly concerned about persistent ageism and some stigma which affects awareness of HIV where older persons are involved. Ageism is experienced in the community as well as at primary health care facilities they attend. In addition, as reported in other studies, our study participants reported that persons aged 50 years and over tend not to be tested for HIV infection, possibly because health professionals view them as sexually inactive and therefore not at risk of infection.^49,50,51,52^ The issue of ageism has been reported as a major obstacle when it comes to HIV and AIDS education and prevention among older persons.^34,53,54,55,56,57,58^ Influenced by societal norms, older people tend to view their sexuality as a private matter, not to be discussed openly even with health professionals.^54,59^ Hence, older persons, in keeping with the perceived attitudes of the public and health professionals, do not view themselves as susceptible to contracting the virus.^32,55,56,60^ Targeting HIV and AIDS prevention programmes for this group is seen as an unproductive exercise.^36,55,60,61^

Even though the focus of our study was on older adults, some participants expressed concern over the youth and the reckless sexual behaviours including a reluctance to use condoms. Concern was expressed, moreover, about risky behaviour engaged in by the youth, particularly those who engage in intergenerational sexual intercourse (young girls with older men), which exposes the young girls, the men and their spouses to a risk of infection. This risk associated with sexual relations between older men and young girls and a reluctance to use a condom has been reported widely.^8,62,63^

Our study participants emphasised the importance of HIV and AIDS educational programmes being tailored specifically for older adults as essential for the prevention of infection in this population. In terms of support and concern for the youth, as reported in another study,^37^ our participants were of the view that education targeted at the older population would impact not only them positively, but the youth as well, as the older generation will then be equipped to reduce their own risk of infection as well as be in a position to act as change agents for the youth.^36,60^ Approaches to the design of education programmes and strategies reported in the literature^32,33,34,37^ suggest similarly that HIV and AIDS education may be more acceptable to older adults, and thus more productive, when they are encouraged to pass on the information to younger, perceivably ‘more at risk’ individuals. The limited number of existing educational models targeted at older adults may well cause health care practitioners and programme developers to adapt the effective formats of HIV and AIDS prevention initiatives designed for younger populations. However, the characteristics and educational needs of young adults and adolescents differ from those of older adults, and the special needs of older adults must be incorporated into new educational and preventive interventions targeted at the older population.^31^

Multilevel approaches are recommended at the individual (micro), network (meso) and systems (macro) levels in HIV and AIDS education programmes for older people.^11,34,37,53,54^ Such approaches will include media awareness campaigns, workshops, publications for the lay public, and individual and couple education. Orel et al. found the distribution of printed public health publications on HIV to older people to be less appropriate and insufficient for this age group.^55^ Such comment would likewise apply to sections of our study population that were found to have a low level of literacy and health literacy.^11^ Older adults elsewhere have suggested that educational programmes be delivered by peers with whom they identify and interact easily.^8,34,64^ In addition, our study participants were of the view that door-to-door campaigns targeted at older household members, involving face-to-face discussions in their nature are more personalised and will be more effective, as older persons pay scant attention in information sessions targeted at large groups. Lieberman reported that education about HIV and AIDS should be delivered to older persons in environments in which they feel most comfortable, to alleviate any anxiety they may experience relating to the subject matter.^65^

Diversity issues, including culture, ethnicity and equitable access to resources, should be incorporated in the design of educational programmes generally – but particularly in the design of HIV and AIDS education models.^33,55,56,66^ The educational information needs to use language that is accessible to the population and where possible to be provided at sites where older persons gather to minimise access as a barrier. In the South African context, seniors’ centres offer an appropriate, supportive space and platform for education on HIV and AIDS prevention. Overall, a senior centre and its conjoint seniors’ club offer an opportunity for socialisation with peers and mutual support in a non-threatening setting. Such a platform can provide a familiar, congenial, non-judgemental, safe and supportive learning space for older members within their community.

### Limitations of the study

The sample of focus group discussants and the number of key informants interviewed in this qualitative study were small, but adequate in terms of the productive and rich information that was yielded. The findings of the study are not transferrable to the multi-cultural older population of South Africa nor necessarily to urban dwelling populations, nor segments of older populations with higher literacy and health literacy levels than the study population. The findings are neither transferable to older persons who do not access a senior centre, in that, a premise of the study, supported by the findings, was the eminent suitability of a seniors’ centre setting for the transmission of educational information. The study was limited in this sense to the membership of only two service centres in rural areas of one of South Africa’s nine provinces. The above said recruitment of the samples at seniors’ centres may have resulted in the participants being better informed about HIV and its prevention, having been exposed to some education on the disease, than non-members of a seniors’ centre in those communities. Participation in the study was voluntary and the majority of the participants were female, which may have added a gender bias to the discussion, but equally may have resulted in frank expression in the discussions. The contribution of the two key informants (centre managers) was of value, as the managers straddled both a seniors’ centre and the community of older persons in the areas and have a keen grasp of the attitudes, behaviour and information needs of this population. Their inputs concurred with and supported the contributions of the participants in the group discussions.

## Conclusions

Older persons in our study had basic knowledge of HIV and AIDS, but perceived they were not as well supported as younger age groups are regarding education about risk, prevention and testing for the virus. A perceived greater focus on the educational needs of younger age groups in this regard perpetuates older persons’ perception that they are not at risk of contracting the virus. Educational programmes should therefore give equivalent attention to older persons’ risk of infection with the HIV. A need for tailored approaches for health professionals to address the disease in this population is similarly indicated. Although our findings are limited to a small sample in two rural areas of the Western Cape Province of South Africa, the study has highlighted a gap in support for the older population regarding HIV and AIDS. Larger studies with a broader case mix are indicated to inform the design of HIV and AIDS educational programmes for this population. When adequately equipped with relevant information, older persons have a potentially strong role to play as change agents in their community in the management and containment of the HIV and AIDS epidemic.

Overall, the importance of the presence of multidisciplinary health care professionals (medical, social work, psychosocial counsellors and media personnel, for example) to address HIV prevention among older persons should be emphasised. Culture-specific programmes are called for, as some individuals may be reluctant to attend programmes on sexuality, discussion thereof being a taboo to them, or, because of peer pressure, not to be seen attending discussions on sexuality.
